# Riemannian geometry-based transfer learning for reducing training time in c-VEP BCIs

**DOI:** 10.1038/s41598-022-14026-y

**Published:** 2022-06-14

**Authors:** Jiahui Ying, Qingguo Wei, Xichen Zhou

**Affiliations:** Department of Electronic Information Engineering, School of Information Engineering, Nanchang University, Nanchang, 330031 China

**Keywords:** Biomedical engineering, Electroencephalography - EEG, Extracellular recording

## Abstract

One of the main problems that a brain-computer interface (BCI) face is that a training stage is required for acquiring training data to calibrate its classification model just before every use. Transfer learning is a promising method for addressing the problem. In this paper, we propose a Riemannian geometry-based transfer learning algorithm for code modulated visual evoked potential (c-VEP)-based BCIs, which can effectively reduce the calibration time without sacrificing the classification accuracy. The algorithm includes the main procedures of log-Euclidean data alignment (LEDA), super-trial construction, covariance matrix estimation, training accuracy-based subject selection (TSS) and minimum distance to mean classification. Among them, the LEDA reduces the difference in data distribution between subjects, whereas the TSS promotes the similarity between a target subject and the source subjects. The resulting performance of transfer learning is improved significantly. Sixteen subjects participated in a c-VEP BCI experiment and the recorded data were used in offline analysis. Leave-one subject-out (LOSO) cross-validation was used to evaluate the proposed algorithm on the data set. The results showed that the algorithm achieved much higher classification accuracy than the subject-specific (baseline) algorithm with the same number of training trials. Equivalently, the algorithm reduces the training time of the BCI at the same performance level and thus facilitates its application in real world.

## Introduction

A brain-computer interface (BCI) is a new communication system that does not rely on the participation of peripheral nerves and muscle tissues. A BCI establishes a direct connection between the brain and a computer or other electronic devices^1,2^. In diverse paradigms of BCIs, visual evoked potential (VEP)-based BCI has recently received increasing attention^3–11^ because it is the BCI most likely to first gain widespread applications in real-world^6,8,10^. Electroencephalogram (EEG) recorded on the scalp is widely used in human BCIs due to its non-invasiveness, easiness to acquire and high time resolution.

VEP signals are responses of the brain to visual stimuli and are mainly generated over the occipital lobes. According to the difference in modulation methods of stimulus signals, VEPs can be divided into time modulated VEP (t-VEP), frequency modulated VEP (f-VEP) and pseudorandom code modulated VEP (c-VEP)^12^. Among them, the BCIs based on the latter two are the most potential BCIs that can achieve very high information transfer rate (ITR), the most significant performance metric for a BCI system^1^.

The first c-VEP BCI was proposed in 1984 and tested 8 years later on an amyotrophic lateral sclerosis (ALS) patient by Sutter^13,14^. The result showed that the subject could write 10–12 words/min. In 2001, Bin et al. developed a 32-target c-VEP BCI with the highest ITR (108 bits/min) among all kinds of BCIs at that time^15^. Subsequently, some research groups reported important progresses in c-VEP BCI studies. Thielen et al.^16^ developed an approach to fully eliminate the tedious training stage, which is able to systematically reduce the training data in a step fashion and ultimately arrive at a calibration-free method for a c-VEP based BCIs. The results showed that the training-less BCI yielded high communication rates in an online spelling task, proving its feasibility for practical use. Wei et al.^17^ proposed a grouping modulation-based c-VEP paradigm that divides all stimulus targets into several groups and targets per group are modulated with a distinct modulation code and its circularly shifting codes. The results indicated that the number of targets and ITR can be increased significantly with the number of groups at the cost of slight sacrifice of classification accuracy. Wittevrongel et al.^18^ introduced a novel decoding algorithm based on spatiotemporal beamforming. They showed that this algorithm significantly outperformed an optimized support vector machine (SVM) classifier for a small number of repetitions of the coding sequence. Riechmann et al.^19^ developed a c-VEP BCI for fulfilling everyday tasks such as navigation or action selection. The study showed that this work supports the notion of c-VEP BCIs as a particularly fast and robust approach suitable for real-world use. Waytowich et al.^20^ presented a novel c-VEP BCI paradigm that realizes spatial decoupling of the targets and flashing stimuli via spatial separation and boundary positioning. Results showed classification accuracies for the non-foveal condition comparable with those for direct-foveal condition for longer observation lengths. Spuler et al.^21^ made use of one-class support vector machines for creating templates and error-related potentials for target recognition and thus the reliability and accuracy of c-VEP BCIs were improved significantly.

Recently, a new family of approaches based on Riemannian geometry has been presented for EEG signal processing^22–24^ Unlike classical methods based on feature vector classification in Euclidean space, these approaches enable classification of EEG signals by direct manipulation of covariance matrices in Riemannian space. They have been successfully applied to BCIs based on motor imagery (MI)^24–26^, f-VEP^27,28^ and P300^29^ and achieved promising results. To the best of our knowledge, they have not been employed in c-VEP BCIs so far. Motivated by the above studies, we developed a Riemannian geometry-based classification frame for classifying c-VEP signals.

Due to the non-stationarity of EEG signals and great variability between subjects, it is necessary to acquire a large amount of training data to calibrate the classification model of a BCI system just before every use of it. This time-consuming training process makes users easy to fatigue and thus severely limits the applications of a BCI in real life. Accordingly, it is desired to develop a reliable method to reduce or even suppress the BCI calibration time while keeping the accuracy within an acceptable range. Transfer learning (TL) is a potential solution for achieving the goal. TL is defined as the ability to apply knowledge learnt in previous tasks or fields to new tasks or fields^30,31^. TL has received widespread attention in the field of BCI for improving the generalization performance of classifiers, such as the BCIs based on MI^32–35^, f-VEP^28,36^ and c-VEP^37^.

In TL, how to effectively transfer knowledge embedded in EEG data from previous subjects (named source subjects hereinafter) to a new subject (named target subject hereinafter) is a crucial problem that needs to be solved. In terms of BCI systems, there are huge differences in data distribution between subjects due to the inter-subject variability. As a result, not EEG data from all source subjects can be transferred as training data to a target subject to construct a good classifier for classifying his/her testing data. To avoid negative transfer, two methods are usually used for increasing the similarity in data distributions between subjects. One is data alignment and the other is source subject selection. The former aims at reducing the difference in data distributions between subjects by aligning data to a common reference point, whereas the latter aims to select those source subjects whose data distributions are more similar to that of the target subject.

It is assumed that a small amount of training data be available from a target subject and the EEG data from some source subjects be used as his/her auxiliary training data. By the aid of a suitable TL algorithm, a robust classification model can be built for the target subject. In this paper, we propose a Riemannian geometry-based TL algorithm for c-VEP BCIs, in which EEG data are aligned by a log-Euclidean mean-based data alignment (LEDA) approach, covariance matrices are estimated from super-trials (a particular type of EEG trials), a subset of source subjects is selected by a training accuracy-based subject selection (TSS) algorithm, and finally a minimum distance to mean (MDM) classifier is constructed with the transferred data from source subjects and training data of the target subject. A c-VEP BCI dataset containing 16 subjects was used for evaluating the proposed algorithm. The results show that the algorithm is able to significantly reduce training time while maintaining high accuracy.

The major contributions of the paper are two-fold: (1) A Riemannian geometry-based classification frame is developed for c-VEP BCIs. The frame enables classification of EEG signals by direct manipulation of covariance matrices and thereby avoids the processing procedures of spatial filtering and feature extraction in classical classification models based on Euclidean space; (2) A transfer learning algorithm is proposed for reducing the training time of c-VEP BCIs without the compromise of classification accuracy. By making use of an LEDA approach and a TSS algorithm, the performance of transfer learning is enhanced significantly compared to the baseline algorithm based on subject specific learning.

## Methods

In this section, we introduce the method for classifying c-VEP signals in Riemannian space and the algorithm for transfer learning in c-VEP BCIs. The former includes the concepts such as sample covariance matrix (SCM), super-trial, covariance matrix estimation and Riemannian geometry, whereas the latter contains the concepts such as instance-based transfer learning, data alignment, source subject selection, and transfer learning-based classification. It is noted that in this paper, (stimulus) target and class are two replaceable words because each stimulus target corresponds uniquely to one category. All the methods were performed in accordance with relevant guidelines and regulations.

### Classifying c-VEP signals in Riemannian space

1. SCM: In BCI experiments, a single trial consists of a task period and a resting period, and is denoted by a segment of time-windowed EEG data in the task period. Let $X_{z} \in R^{{N_{c} \times N_{t} }}$ be a single-trial bandpass filtered EEG signal with a zero mean, where $z \in \{ 1,2, \ldots ,Z\}$, $N_{c}$ and $N_{t}$ denote the indices of $Z$ classes/targets, the number of electrode channels and the number of sampling points respectively. SCM is commonly used for optimizing spatial filters and classifiers in conventional machine learning in Euclidean space. The SCM of an EEG signal $X_{z}$ is usually estimated as follows
  1 $$ P_{z} = \frac{1}{{N_{t} - 1}}X_{z} X_{z}^{T} {\kern 1pt} {\kern 1pt} \in R^{{N_{c} \times N_{c} }} $$
  Each diagonal element of $P_{z}$ holds the variance of the signal at one electrode and each of its off-diagonal elements holds the covariance between one pair of electrodes. Thereby, the SCM contains all spatial information of the EEG signal, particularly its second-order statistics.
2. Creating super-trials for c-VEP signals: For the BCI paradigm based on MI, the SCM suffices for discriminating EEG trials of different classes, since MI-based trials for different classes do induce different spatial patterns. For a typical BCI paradigm based on c-VEP, however, all stimulus targets are encoded by the same modulation sequence with different time lags, and thus c-VEP trials can only be differentiated by time lags or phase differences. The SCM of an EEG trial is not useful for classifying c-VEP signals because it does not possess any temporal information at all.

In order to classify the c-VEP signals in Riemannian space, it is necessary to embed the temporal information into a covariance matrix^23^. To this end, we need to first create a super-trial for a training trial

2 $$ X_{z}^{^{\prime}} = [\overline{X}_{1} ;{\kern 1pt} {\kern 1pt} {\kern 1pt} {\kern 1pt} \overline{X}_{2} ;{\kern 1pt} {\kern 1pt} {\kern 1pt} \cdots ;{\kern 1pt} {\kern 1pt} {\kern 1pt} \overline{X}_{Z} ;{\kern 1pt} {\kern 1pt} {\kern 1pt} X_{z} ] \in R^{{N_{c} (Z + 1) \times N_{t} }} $$

where $\overline{X}_{1} ,{\kern 1pt} {\kern 1pt} {\kern 1pt} \overline{X}_{2} ,{\kern 1pt} {\kern 1pt} {\kern 1pt} \ldots ,\overline{X}_{Z}$ are the template signals of $Z$ targets yielded by averaging the EEG trials of the same target, and $X_{z}$ is an EEG trial from the target $z$. The SCM of the super-trial can be calculated as

3 $$ P_{z}^{^{\prime}} = \frac{1}{{N_{t} - 1}}X_{z}^{^{\prime}} (X_{z}^{^{\prime}} {\kern 1pt} )^{T} {\kern 1pt} \in R^{{N_{c} (Z + 1) \times N_{c} (Z + 1)}} $$

where the dimension of $P_{z}^{^{\prime}}$ is $N_{c} (Z + 1) \times N_{c} (Z + 1)$, increasing significantly compared to that of $P_{z}$ in Eq. (1). The class information is embedded into covariance matrix $P_{z}^{^{\prime}}$, which can be applied for classifying c-VEP signals accordingly. Given an unlabelled testing trial $X$, its super-trial and corresponding SCM can be computed using Eq. (2) by replacing $X_{z}$ with $X$ and using Eq. (3) respectively.

(3) Covariance matrix estimation: Since covariance matrices must be accurate, symmetric positive definite (SPD), and well-conditioned (this property requires that the ratio of the largest singular value to the smallest singular value cannot be too large), the choice of a covariance matrix estimator is very important. Although the SCM estimator formulated in Eq. (1) is computationally efficient, the covariance matrix estimated by it often fails to meet the above requirements, especially when the size of a covariance matrix is large such as $P_{z}^{^{\prime}}$ in Eq. (3). To address the problem, shrinkage estimators^27^ were developed as a weighted sum of the SCM $P_{z}^{^{\prime}}$ and a target covariance matrix $\Gamma$, which is chosen to close to the identity matrix. The shrinkage estimators are defined as
  4 $$ P_{sh} = \lambda \Gamma + (1 - \lambda )P_{z}^{^{\prime}} $$
  where $0 \le \lambda < 1$ is a weighting coefficient. The shrinkage estimators differ in their definition of target covariance matrix $\Gamma$. Ledoit and Wolf^38^ proposed $\Gamma = vI_{{N_{c} }}$ with $v = tr(P_{z}^{^{\prime}} )$; Blankertz^39^ also defined $\Gamma = vI_{{N_{c} }}$ but with $v = tr(P_{z}^{^{\prime}} )/N_{c}$; Schafer and Strimmer^40^ presented several methods for defining $\Gamma$ relying on the SCM $P_{z}^{^{\prime}}$. The last shrinkage estimator mentioned above was used in this study because it provides a good tradeoff between performance and operational speed.

(4) Riemannian geometry: In conventional BCI systems, many methods for feature extraction and classification developed in Euclidean space such as common spatial pattern (CSP), canonical correlation analysis (CCA) and linear discriminant analysis (LDA), are based on the SCMs of EEG signals. The SCM of an EEG trial, however, is actually an SPD matrix and thus lies in a Riemannian manifold, a subset of Euclidean space. Because the space of SPD matrices endowed with Riemannian distance is a differentiable Riemannian manifold^41,42^, the notions from Riemannian geometry such as Riemannian distance and Riemannian mean, can be used to analyze and classify SCMs.

Denote by ${\mathcal{S}}(n) = \{ S \in M(n),S^{T} = S\}$ the space of all $n \times n$ symmetric matrices in the space of square real matrices $M(n)$ and $P(n) = \{ P \in S(n),u^{T} Pu > 0,\forall u \in R^{n} \}$ the set of all $n \times n$ SPD matrices. As for the Riemannian distance of two SPD matrices, there are several different definitions in literature^43^, the most commonly used two of which are affine-invariant distance and log-Euclidean distance. The expression of the former is as follows

5 $$ d_{AI} (P_{1} ,P_{2} ) = ||\log (P_{1}^{ - 1} P_{2} )||_{F} = \sqrt {\sum\nolimits_{e = 1}^{{N_{e} }} {\log^{2} \lambda_{e} } } $$

where $\lambda_{e} ,{\kern 1pt} {\kern 1pt} {\kern 1pt} e = 1,2, \ldots ,N_{e}$ is the *e*th eigenvalue of matrix $P_{1}^{ - 1} P_{2}$; whereas that of the latter is as follows

6 $$ d_{LE} (P_{1} ,P_{2} ) = ||\log (P_{1} ) - \log (P_{2} )||_{F} {\kern 1pt} $$

Since $P_{1}$ and $P_{2}$ are SPD matrices, the two types of Riemannian distances have an important property termed as congruence invariance

7 $$ d_{B} \left( {P_{1} ,P_{2} } \right) = d_{B} \left( {UP_{1} U^{T} ,UP_{2} U^{T} } \right),{\kern 1pt} {\kern 1pt} {\kern 1pt} B = AI{\kern 1pt} {\kern 1pt} {\kern 1pt} or{\kern 1pt} {\kern 1pt} {\kern 1pt} LE $$

where $U$ denotes an invertible and orthogonal matrix. Because of the excellent property, the linear transformation of two SPD matrices will not change their relative distance in the Riemannian space.

The Riemannian mean of multiple SPD matrices is defined by their Riemannian distance with a variational approach^44^. Since $P_{n} ,n = 1,2, \ldots ,N$ lies in a Riemannian manifold of non-positive curvature, the Riemannian mean is defined as the matrix that minimizes the sum of the squared Riemannian distances

8 $$ \overline{P}_{R} \left( {P_{1} , \ldots ,P_{N} } \right) = \mathop {\arg \min }\limits_{P \in P\left( n \right)} \sum\limits_{i = 1}^{N} {d_{R}^{2} } \left( {P_{i} ,P} \right) $$

The existence and unicity of the Riemannian mean were proved in Ref.^45^. When the affine-invariant distance metric is used as Riemannian distance, an explicit solution exists only for $N = 2$, where it coincides with the middle point of the geodesic connecting the two SPD matrices. For $N > 2$, a solution can be found iteratively, and several different algorithms have been developed in Ref.^46^; When the log-Euclidean distance is used as Riemannian distance, an explicit solution exists and can be formulated as

9 $$ \overline{P}_{LE} \left( {P_{1} , \ldots ,P_{N} } \right) = \mathop {\arg \min }\limits_{P \in P(n)} \sum\limits_{i = 1}^{N} {d_{LE}^{2} (P_{i} ,P) = } \exp \left( {\sum\limits_{i = 1}^{N} {\log (P_{i} )} } \right) $$

In Riemannian space, SPD matrices function as feature signals and the decoding of EEG signals is able to directly operate on them. There are two methods for achieving the task: One is the minimum distance to mean (MDM) classifier proposed by Congedo et al.^23^ and the other is tangent space LDA (TSLDA). The former classifies an EEG trial by comparing the Riemannian distances of its covariance matrix to the Riemannian means of all classes, whereas the latter first maps covariance matrices onto tangent space, then the matrices are vectorized, and finally the feature vectors are classified with conventional algorithms from the Euclidean space. This study adopted the first method due to the simplicity of its concept.

With the concepts of distance and mean in Riemannian space, an MDM classifier can be used to classify EEG trials by directly manipulating their covariance matrices. Thereby, the classification frame avoids spatial filtering and feature extraction in conventional classification algorithm based on Euclidean geometry. In a c-VEP BCI, the training data from the reference target can be shifted to obtain the training data of other targets according to their time lags in modulation codes. Using labelled training trials, we can calculate the mean covariance matrix of each class $z$, denoted as $M_{z} ,{\kern 1pt} {\kern 1pt} {\kern 1pt} z = 1,2, \ldots ,Z$. Then an unlabelled testing trial is assigned to the class with the closest mean, which means that the distance between the covariance matrix of the testing trial and the $M_{z}$ of the assigned class $z$ is the smallest. The detail of this classification algorithm can be referred to Ref.^23^.

### Transfer learning for c-VEP BCIs

1. Instance-based transfer learning: Transfer learning (TL) is an important branch of machine learning^30,31^. In TL, a domain ${\mathcal{D}}$ consists of a feature space ${\mathcal{X}}$ and its marginal probability distribution $P(X)$, i.e., ${\mathcal{D}} = \{ {\mathcal{X}},{\kern 1pt} {\kern 1pt} {\kern 1pt} P(X)\}$, where $X \in {\mathcal{X}}$. Two domains ${\mathcal{D}}_{S}$ and ${\mathcal{D}}_{T}$ are different if ${\mathcal{X}}_{S} \ne {\mathcal{X}}_{T}$ and/or $P_{S} (X) \ne P_{T} (X)$. A task ${\mathcal{T}}$ consists of a label space ${\mathcal{Y}}$ and a conditional probability distribution $Q({\mathcal{Y}}|X)$. Two tasks ${\mathcal{T}}_{S}$ and ${\mathcal{T}}_{T}$ are different if ${\mathcal{Y}}_{S} \ne {\mathcal{Y}}_{T}$ and/or $Q_{S} ({\mathcal{Y}}|X) \ne Q_{T} ({\mathcal{Y}}|X)$. TL uses the similarity among data, tasks, or models to transfer the data or knowledge from one domain (termed source domain) to help solve the task in another domain (termed target domain).
  In most BCI applications, the feature space and label space are usually assumed the same for source and target domains because the electrode channels used for recording EEG data and the mental tasks the subjects fulfilled are the same, but their marginal and conditional probability distributions are different due to the non-stationarity of EEG signals. There are three approaches for transfer learning in the BCI field^31^, named feature representation transfer, instance transfer and classifier transfer. Based on the second approach, this study made use of the labelled EEG trials of source subjects to help the target subject with a small number of labelled training data calibrate his/her classification model.
2. Data alignment: Due to the randomness and non-stationarity of EEG signals, there are big differences in data distribution between subjects. Thereby, to transfer data from a subject to another, it is necessary to reduce the difference in data distribution between them prior to transferring data. Data alignment (DA) is an efficient method for achieving the goal^47–49^. DA aligns either the EEG trials or their covariance matrices from different domains (i.e., subjects or sessions) to a common reference and thus reduces the marginal probability distribution shift of different domains. DA can be used as a preprocessing step to enhance similarity between EEG trials of different subjects. Several DA approaches exist in literature such as Riemannian alignment (RA)^47^, Euclidean alignment (EA)^48^ and online pre-alignment (OPS)^49^, depending upon how the reference matrix is defined. LEDA, whose reference matrix is derived from log-Euclidean mean, is applied in the TL framework because it outperforms other approaches for c-VEP data.

LEDA can be used in either supervised or unsupervised manner. For testing data from the target subject, LEDA is unsupervised since they do not have any labelled information. LEDA calculates the log-Euclidean mean $M_{LE}$ of the covariance matrices from all classes using Eq. (9), and the reference matrix is calculated as $M_{ref} = P_{LE}^{ - 1/2}$. A single-trial EEG signal (or rather a super-trial) $X_{i}$ is aligned as

10 $$ X_{i}^{^{\prime}} = M_{ref} X_{i} $$

For the EEG data from source subjects and a few labelled training data from the target subject, LEDA can be used in a supervised manner. The log-Euclidean mean $M_{LE,z}$ of covariance matrices can be calculated using all EEG trials belonging to the same class $z \in \{ 1,2, \ldots ,Z\}$, and the reference matrix is $M_{{ref,{\kern 1pt} z}} = M_{LE,z}^{ - 1/2}$. A single-trial EEG signal $X_{i,z}$ is aligned as follows

11 $$ X_{i,z}^{^{\prime}} = M_{ref,z} X_{i,z} $$

DA does not change the relative distance between any two covariance matrices in a data set to be aligned because of the property of congruence invariance in Eq. (7). After LEDA, marginal probability distribution shift of EEG data between subjects is reduced, and resulting EEG trials are whitened accordingly. The distribution of EEG trials from different subjects tends to be consistent, which is conducive to construction of the classifier of a target subject.

(3) Source subject selection: Subject selection (SS) aims to determine an optimal subset of source subjects for a target subject to ensure the highest classification accuracy of his/her testing data with the help of EEG data from the chosen source subjects. Lotte et al.^50^ used the sequential forward floating search algorithm to select the subjects to be added to or removed from the current subject subset in order to maximize the accuracy for the target subject. Qi et al.^51^ proposed to use Riemannian distance to select samples from the existing subject database to help construction of the classifier. Giles et al.^52^ proposed a new similarity measurement algorithm, called Jensen Shannon ratio (JSR), which is used to compare the data of the target subject with the existing subject database, and to select the previously calibrated BCI model with the highest similarity with the target subject as the target model of BCI. Azab et al.^32^ proposed a similarity measurement method based on Kullback–Leibler divergence (KL), which is used to measure the similarity between two feature spaces. Despite these methods, SS remains an open and tough problem.

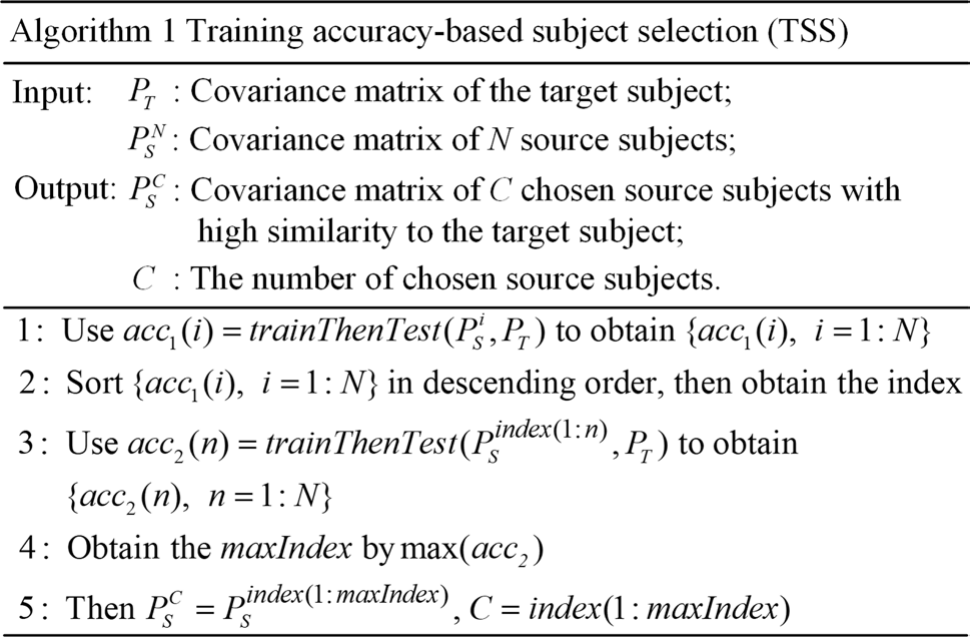


We propose a training accuracy-based subject selection (TSS) algorithm shown in Algorithm 1, where the function $acc = train\;Then\;Test(P_{S}^{N} ,P_{T} )$ returns the accuracy obtained when the MDM is trained on the data set $P_{S}^{N}$ from $N$ source subjects and then is used to classify training data set $P_{T}$ from the target subject. All source subjects are sorted by their classification accuracies. The first $C$ source subjects are selected as transferred subjects, if their EEG data are pooled together to re-train an MDM classifier and it achieves the highest accuracy on data set $P_{T}$ among all subsets of source subjects. The processing procedures of the algorithm are summarized as follows: All source subjects for a target subject are first sorted according to their classification accuracies by using the EEG data from a single source subject as training data and classifying the training data from the target subject. Then c (from 1 to 15) source subjects are sequentially extracted from sorting list and their EEG data are collectively used for creating a classification model, which is used to classify the training data of the target subject. Finally, the C is decided as the c value that yields the highest classification accuracy.

**Figure 1:**
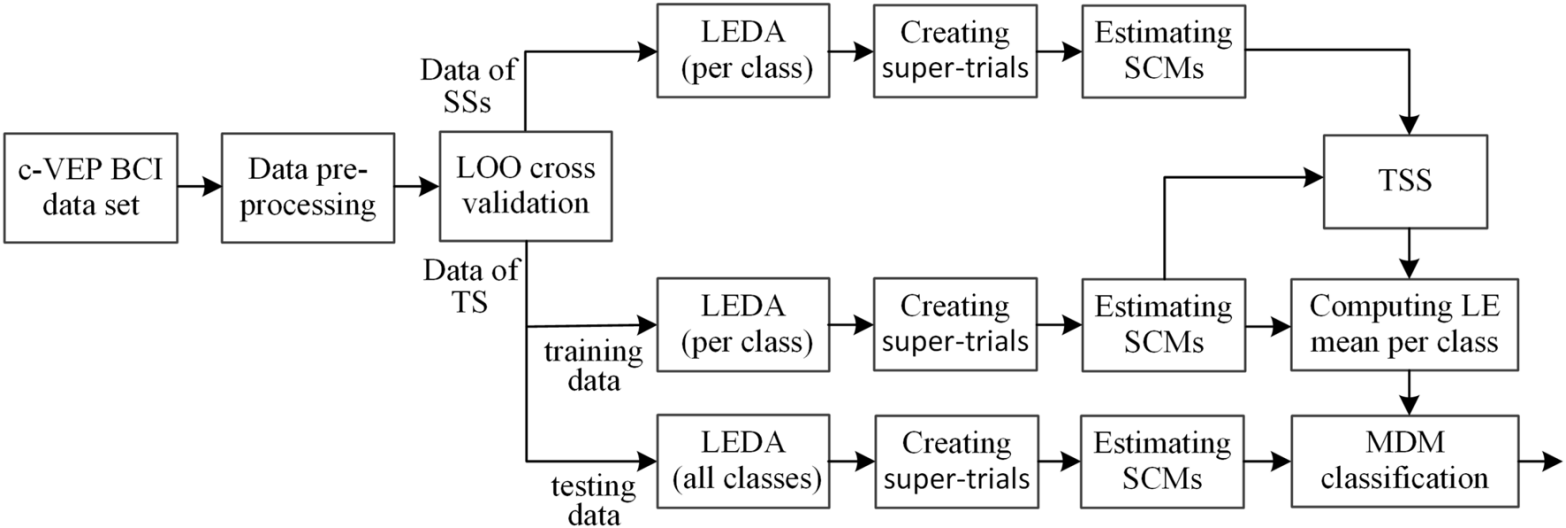
Processing flowchart of the Riemannian geometry-based transfer learning in c-VEP BCIs. The meanings of the acronyms in the figure are as follows. LOO: leave-one subject-out; SSs: source subjects; TS: target subject; LE(DA): log-Euclidean (data alignment); SCMs: sample covariance matrices; TSS: training accuracy-based subject selection; MDM: minimum distance to mean.

(4) Transfer learning-based classification: Given a c-VEP BCI data set, the EEG data from each subject are first preprocessed by bandpass filtering and data segmenting into single-trial signals. The bandpass filtering is done between 2 and 30 Hz via a Butterworth IIR filter of order 8. A leave-one subject-out (LOO) cross validation strategy is used to divide all subjects in the data set into the target subject and source subjects, i.e., each subject in the data set acts as the target subject once, and the remaining subjects act as the source subjects. Then the preprocessed EEG trials from a subject are aligned with LEDA in either supervised or unsupervised manner depending on the types of the trials. For each source subject, the EEG trials function as training data and are aligned by class; for the target subject, the training trials are also aligned by class, whereas the testing trials from all classes are aligned as a whole. Next, for an aligned trial, a super-trial is created with template signals of all targets and an SCM is estimated using the specified shrinkage estimator. Next, the transferred subjects are selected from source subjects according to the TSS algorithm. Finally, the EEG trials of the transferred subjects and the training trials of the target subject are pooled together as overall training data and are employed to computing the log-Euclidean means of all classes, which are utilized to classify the SCMs of testing trials from the target subject with an MDM classifier. The algorithmic flowchart of the transfer learning-based c-VEP BCI classification in Riemannian space is shown in Fig. 1.

## BCI experiment

### Subjects

An offline experiment was designed for the c-VEP BCI. Sixteen healthy subjects (7 females, aged 21 ~ 26 years, mean age: 23 years) with normal or corrected to normal vision participated in the experiment. None of them had a previous history of epilepsy or seizures, which can be induced by flashing stimuli. Six subjects participated in a c-VEP BCI experiment before and were familiar with the experimental setting. Each subject was asked to read and sign an informed consent form before the experiment. The study was approved by the Human Research and Ethics Committee, Nanchang University. After the experiment, these subjects were paid for their contribution to the study.

### Target modulation

**Figure 2:**
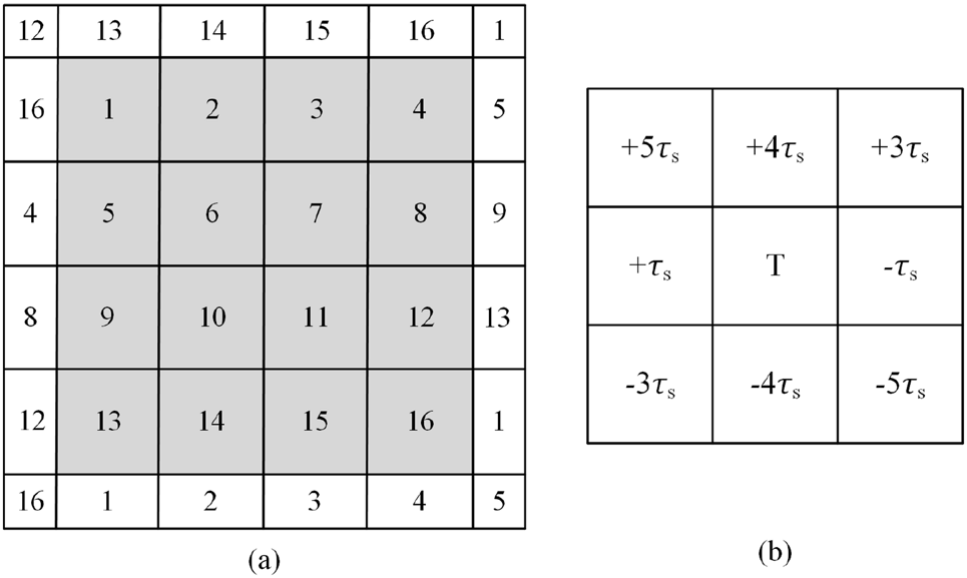
(**a**) The visual stimulator used in the study. It consists of 16 target stimuli in the gray area and 20 complementary non-target stimuli in the white area. The 16 target stimuli are modulated by a 63-bit M sequence and its circularly shifting sequences. The target and non-target stimuli tagged with the same numbers are modulated by the same modulation code. (**b**) The principle of equivalent neighbors is used in design of the stimulator. Each target T and the eight adjacent targets around it maintain a fixed time delay relationship. $\tau_{s}$ (4 bits in the study) is the time lag of modulation codes between two adjacent target stimuli.

A visual stimulator comprising 16 target stimuli is shown in Fig. 2a, the design principle of which is similar to that of Bin et al.^12^. The stimulator was presented on an LCD monitor. Principle of equivalent neighbors was adopted for designing the stimulator. 16 target stimuli and 20 complementary non-target stimuli are arranged in grey and white area respectively. All these stimuli are tightly placed without intervals between them. The 16 target stimuli are modulated by a 63-bit pseudorandom M sequence and its circularly shifting sequences. The 63-bit M sequence corresponds to a stimulus period of P = 63/60 = 1.05 s for the screen refresh rate of 60 Hz, and is circularly shifted integral multiples of 4 bits to generate 16 sequences of different phases used for modulating the 16 target stimuli. The target and non-target stimuli tagged with the same numbers are modulated by the same sequence. The purpose of these non-target stimuli is to ensure that the target stimuli have equivalent neighbors in the directions of left, right, up, down and diagonals, i.e. the time lags between modulation codes of target stimuli and their neighbors in same directions are the same, as shown in Fig. 2b.

### Experimental setup

The experimental system consists of a personal computer (Lenovo China) operated under a Windows 7 system and an EEG amplifier (a Synamps2 system, Neuroscan Inc.) with 64 EEG channels. The computer has a 24-in. liquid crystal display (LCD) monitor and a parallel port linked to the amplifier. The LCD monitor has a refresh rate of 60 Hz and a resolution of 1920 × 1080 pixels. Stimulus presentation was operated in the PC and controlled by the stimulus program developed under Visual C++ 9.0 platform. DirectX (Microsoft Inc.) was employed to ensure the stability of frame-based rendering of stimuli. Event trigger signals yielded by the stimulus program were sent from the parallel port of the computer to the EEG amplifier and recorded on an event channel for synchronizing stimulus presentation and EEG data recordings. Each target stimulus was rendered within a square of 140 × 140 pixels.

During the experiment, all stimuli were flashed simultaneously and periodically according to their modulation codes. Each subject was seated in a comfortable chair in an unshielded, dimly lit room, approximately 60 cm away from the monitor. They were instructed to focus attention to an intended target, gaze at its center and not to blink as much as possible. Nine Ag/AgCl electrodes over the occipital lobes (P3, Pz, P4, PO7, POz, PO8, O1, Oz, O2) in line with international 10/20 system were used for recording EEG data. The reference electrode was positioned at the vertex. Electrode impedances were kept below 10 kΩ during the experiment. The EEG signals were digitized at a sampling rate of 1000 Hz.

### Data acquisition

This experiment was divided into two phases: training and testing. The purpose of the training stage was to acquire training data of a reference target. Any one of the 16 targets could be specified as the reference target, which was the 11th target in the study. The subject should fixate continuously at the reference target for 100 stimulus cycles (i.e. 100 trials of one stimulus cycle). In testing stage, the subject was asked to complete the testing task of all 16 targets, which appeared in a random order. The subject was required to fixate continuously on the current target for five stimulus cycles (i.e., five trials), and subsequently, the test of next target began immediately. Each test started with a prompting duration of 1 s, in which the target turned red and the subject should shift his/her gaze to the target as soon as possible. After the cue duration was over, the red target cue was switched to a small cycle at bottom right corner of the target so that the subject found it easily.

## Results

The difference in data distribution between the raw data and the aligned data is visualized in 2-dimensional space to examine the effect of LEDA. The following four Riemannian geometry-based algorithms are compared using the first algorithm as baseline: (1) subject-specific learning (SSL); (2) transfer learning using EEG data of all source subjects without data alignment (TL-ASS); (3) transfer learning using EEG data of all source subjects with LEDA (TL-LEDA-ASS); and (4) transfer learning using EEG data of chosen source subjects by TSS with LEDA (TL-LEDA-TSS). It is noted that for each source subject, only testing data (5 trials per target) are used for transfer learning because each subject had only training data from one stimulus target. The proposed classification algorithm is evaluated by classification accuracy and ITR based on varying lengths of data, numbers of channels and numbers of training trials. The ITR in bits/minute defined by Wolpaw et al.^1^ is calculated as follows

12 $$ ITR = \left( {\log_{2} M + P\log_{2} P + (1 - P)\log_{2} \left[ {\frac{1 - P}{{M - 1}}} \right]} \right)*\left( \frac{60}{T} \right) $$

where *M* is the number of targets, *P* is the detection accuracy of targets, and *T* in seconds/selection is the average time for a selection. For the estimation of ITRs, the time of 1 s for gaze-shifting was included in target selection.

This definition for ITR is a simplified computational model based on Shannon channel theory under several assumptions and the most widely used metric to assess the overall performance of a BCI system. Although several scholars, e.g., Yuan et al.^53^, pointed out its shortcomings and proposed some improvements, the vast majority of studies still use this definition to measure the performance of BCI systems so far.

### Data visualization

**Figure 3:**
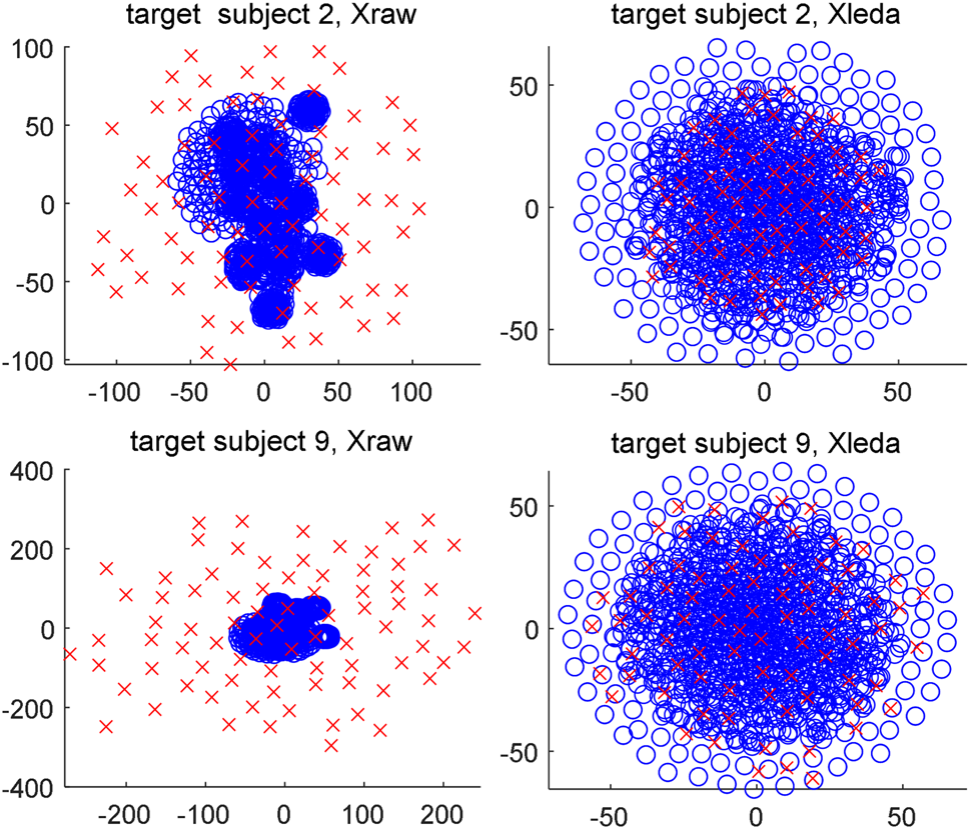
t-SNE visualization of the distribution of raw data (Xraw, the first column) and aligned data by LEDA (Xleda, the second column). The red symbols ‘ × ’ denote the EEG trials from the target subject (16 × 5 = 80 trials), whereas the blue symbols ‘o’ stand for the EEG trials from all source subjects (80 × 15 = 1200 trials).

t-distributed stochastic neighbor embedding (t-SNE)^54^ is a commonly used method for data dimensionality reduction and visualization. High-dimensional data can be projected to a 2- or 3-dimensional space by t-SNE and their characteristics in distribution are observed in the space. We visualize whether LEDA can decrease the difference in data distributions between the target subject and source subjects. Figure 3 shows two examples of 2-dimensional distributions of the raw data and the aligned data by LEDA. In each subplot, the red symbols " × " indicate the EEG trials of the target subject, whereas the blue symbols "o" denote the EEG trials of all source subjects. From the figure, it is clearly observed that for the raw data, the data distributions of the target subject and the source subjects are totally different, whereas for the aligned data, they tend to be consistent. Thereby, the data alignment by LEDA is very conducive to decreasing the difference in data distribution between subjects and subsequent transfer learning for c-VEP BCIs.

### Transferring data from a single source subject

**Figure 4:**
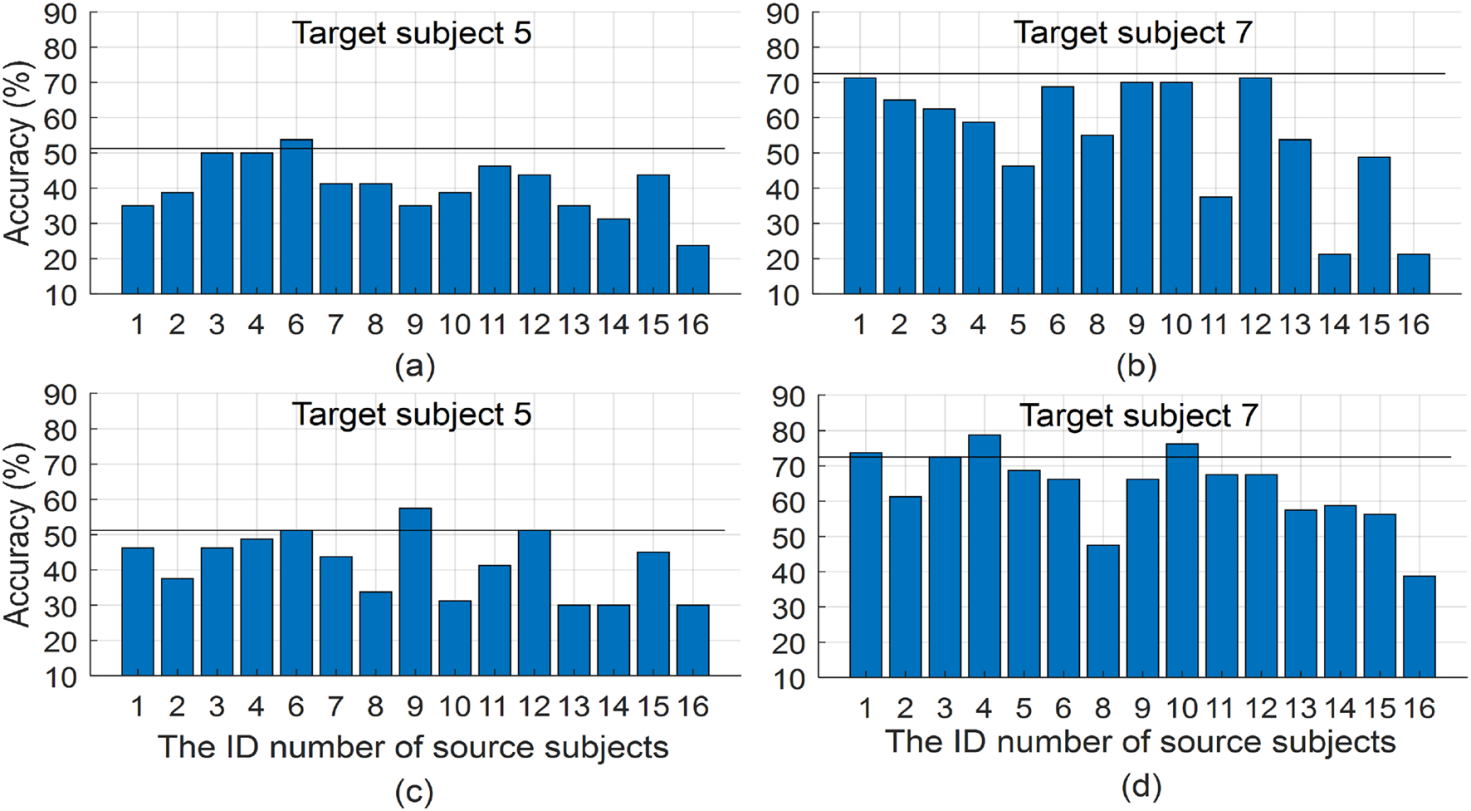
Classification accuracies of two target subjects (#5 and #7) yielded by the two transfer learning algorithms TL (**a**,**b**) and TL-LEDA (**c**,**d**) with the EEG trials of only one source subject transferred to the target subject. The sold line in each subplot stands for the accuracy yielded by SSL (baseline) algorithm.

Figure 4 depicts the classification accuracies of two target subjects (#5 and #7) yielded by the two transfer learning algorithms TL (the first row) and TL-LEDA (the second row) with the EEG trials (5 trials per class) of only one source subject transferred to the target subject. The accuracy achieved by SSL algorithm is also shown with a sold line as a baseline in each subplot. The same 5 training trials per stimulus target (obtained by shifting the 5 trials of the reference target) from the target subject were used for building classification model in both transfer algorithms and SSL algorithm. From the figure, it is easily observed that whether the EEG trials are aligned or not, transferring EEG data between different pairs of source-target subjects, classification accuracies differ substantially. In most cases, such a transfer was negative because it deteriorated the accuracy. However, data distributions of some sources are complementary and their overall distribution may be more similar to that of the target subject. To avoid negative transfers, it is necessary to select those relevant source subjects whose total data distribution is more similar to that of the target subject.

### Transferring data from multiple source subjects

**Figure 5:**
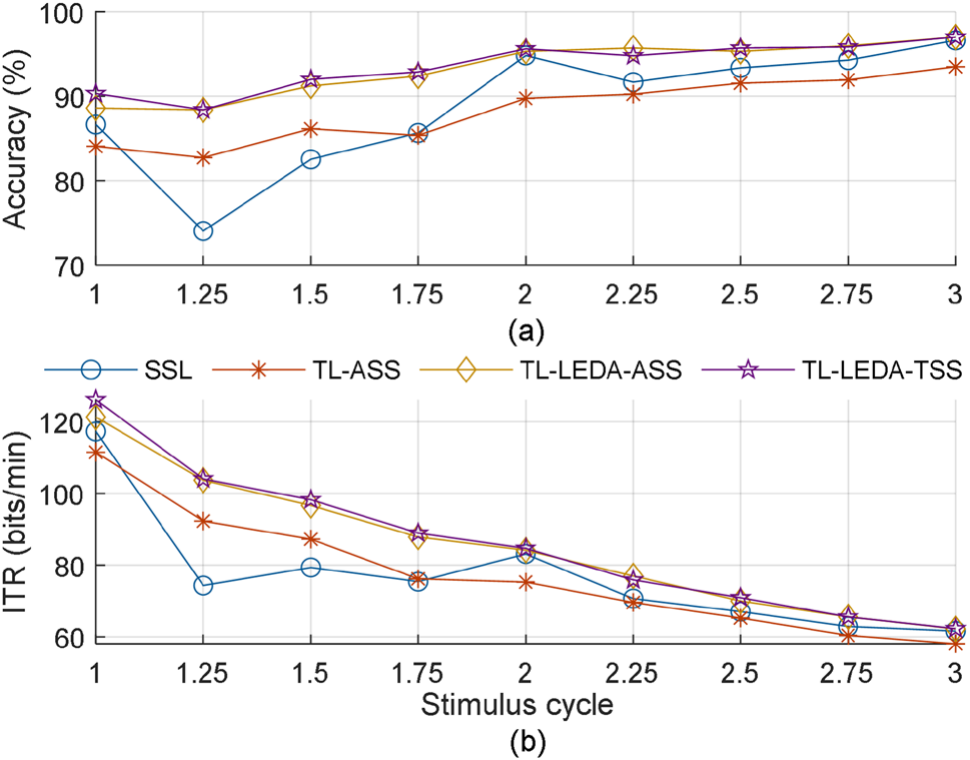
Relationship between the averaged accuracy (**a**) and ITR (**b**) of the four algorithms (SSL, TL-ASS, TL-LEDA-ASS and TL-LEDA-TSS) across subjects and the data length used for target recognition. The data length is denoted as the multiple of the stimulus cycle.

**Figure 6:**
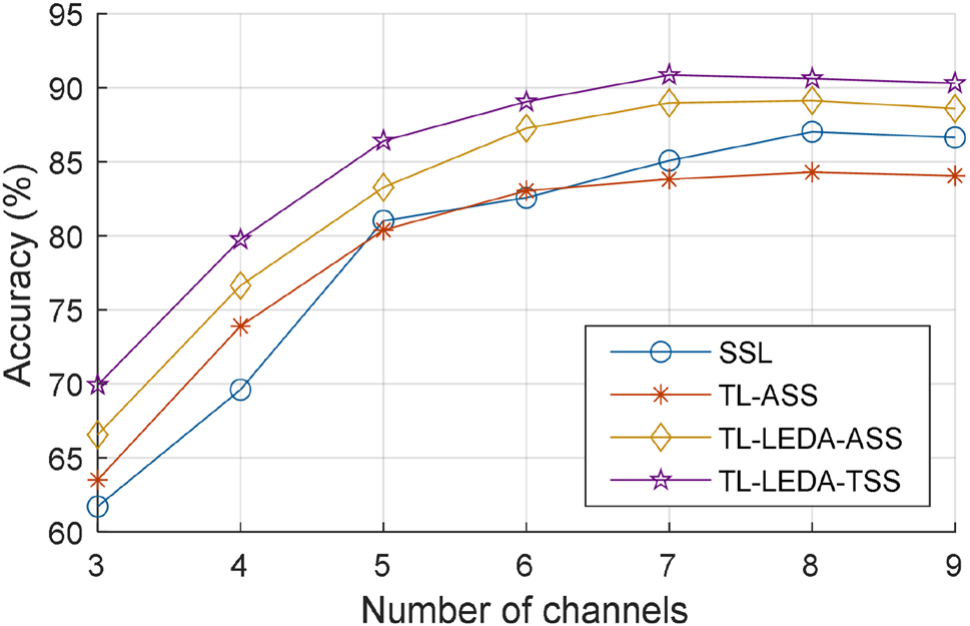
Relationship between the averaged accuracy across subjects and the number of channels used for target recognition for the four algorithms SSL, TL-ASS, TL-LEDA-ASS and TL-LEDA-TSS.

**Figure 7:**
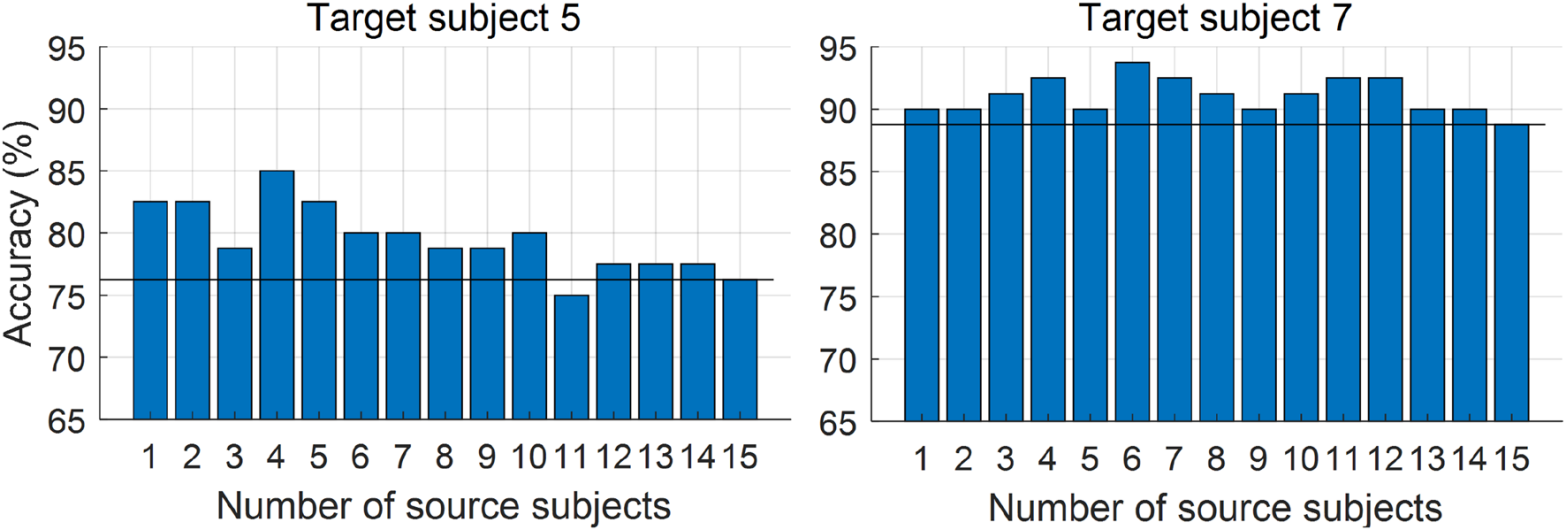
The classification accuracies of the two target subjects (#5 and #7) change with the number of chosen source subjects. The solid line in each subplot denotes the accuracy of SSL (baseline) algorithm for comparison.

1. Data length: Fig. 5 illustrates the relationship between the averaged accuracy and ITR of the four algorithms (SSL, TL-ASS, TL-LEDA-ASS and TL-LEDA-TSS) across subjects and the data length used for target recognition. The data length is denoted as multiple of the stimulus cycle, and 9 different multiples are taken from 1 to 3 with an interval of 0.25, i.e. $k = 1,1.25, \ldots ,3$. The number of training trials and the number of channels used for classification are 20 and 9 respectively. It is easily seen from the figure that although the overall trend of the accuracy yielded by each algorithm is to increase with data length, the ITR indeed decreases monotonically. Statistical analysis based on paired t-tests showed that the ITRs of the four algorithms at one stimulus cycle are significantly higher than those at all other data lengths with $p < 0.001$. Therefore, the data length of one stimulus cycle was used in the following analysis.
2. The number of channels: Fig. 6 shows the relationship between the averaged classification accuracy across subjects and the number of channels for each of the four algorithms SSL, TL-ASS, TL-LEDA-ASS and TL-LEDA-TSS. In the study, the nine channels (P3, Pz, P4, PO7, POz, PO8, O1, Oz, O2) were used to record EEG data and kept sequentially in the dataset. The number of channels used for classification was taken from 3 to 9 with the step length of 1. To simplify the analysis, the channels were selected sequentially according to their order in the dataset. The number of training trials used for classification is 20. As shown in the figure, accuracies of all the four algorithms first rise rapidly, then slow down and fall after reaching the maxima. For SSL, TL-ASS and TL-LEDA-ASS, the maximal accuracies were achieved at 8 channels, whereas for TL-LEDA-TSS, that was derived at 7 channels. Paired t-tests showed that TL-LEDA-TSS has no significant differences in accuracy between 7 and 8 channels with $p = 0.10$. Thus, 8 channels were used for following analysis.
3. The number of chosen source subjects: In this study, we screened the source subjects with Algorithm 1. It is noted that the testing data set $P_{T}$ of the target subject was derived from training data of all targets obtained by shifting those of the reference target, whereas the training data set $P_{S}^{N}$ of $N$ source subjects was derived from the testing data, i.e., 5 trials per target. Figure 7 shows the classification accuracies of the two target subjects (#5 and #7 as examples) varying with the number of source subjects used for transferring data. The number of training trials is taken as 20. The solid line in each subplot denotes the accuracy of SSL (baseline) algorithm for comparison. Obviously, the accuracy does not increase monotonically with the number of source subjects, but fluctuates up and down near the baseline accuracy. Based on the TL-LEDA-TSS algorithm, the number of chosen source subjects yielding the highest accuracy is 4 and 6 for the two target subjects respectively.

Table 1 lists the classification accuracies of the 16 target subjects under three conditions: Acc1—the maximum accuracy achieved actually; Acc2—the minimum accuracy achieved actually; Acc3—the accuracy yielded by TL-LEDA-TSS algorithm. The corresponding number of transferred source subjects is shown in parentheses. As shown in the table, the accuracy of each target subject yielded by TL-LEDA-TSS algorithm (Acc3) is not identical to the maximum accuracy achieved actually (Acc1), so is their corresponding numbers of chosen source subjects. The mean accuracy of all target subjects achieved by the algorithm is lower than Acc1, meaning that this TSS algorithm is not optimal. However, the TSS algorithm achieved a near-optimal mean accuracy much higher than the minimum accuracy achieved actually. It is better to show which source subjects were selected for a target subject. We ignored this problem for sparing space.

**Figure 8:**
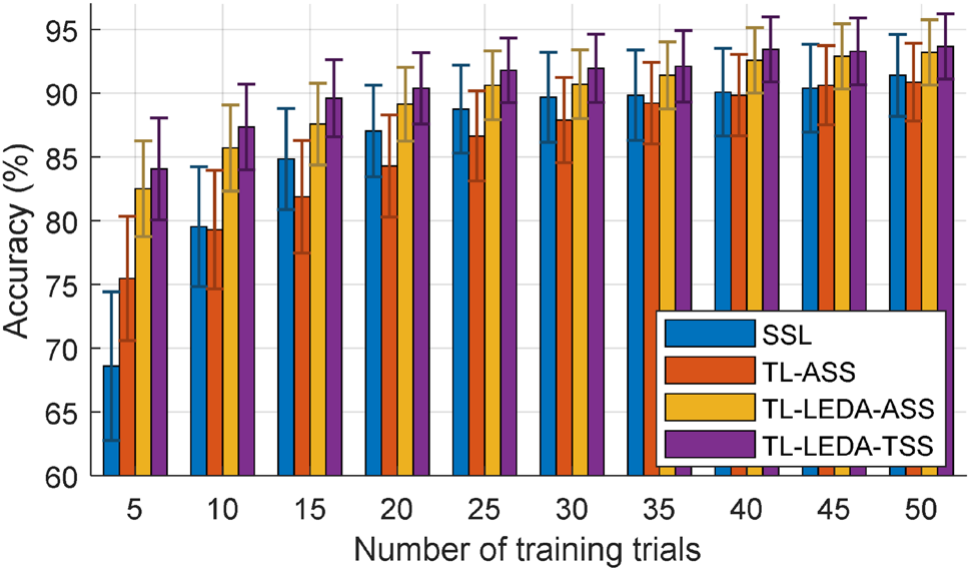
Averaged classification accuracies across all subjects of the four algorithms varying with the number of training trials, which ranges from 5 to 50 with the interval of 5 trials. The error bars represent the standard error.

(4) The number of training trials: Fig. 8 illustrates the averaged classification accuracies across all subjects of the three transfer learning algorithms varying with the number of training trials, which ranges from 5 to 50 with the interval of 5 trials, i.e., $N_{tr} = 5,10, \ldots ,50$. The classification accuracy of SSL algorithm is shown as baseline for comparison. Clearly, the accuracy of each algorithm increases consistently with the number of training trials, but the increase slows down as it gets bigger. The accuracies of TL-ASS are always lower than those of SSL except for $N_{tr} = 5$ trials, meaning that without additional processing of c-VEP data, directly transferring them from source domains to a target domain is generally not feasible.

**Table 1:** The classification accuracies (%) of the 16 target subjects (TS) and their mean (M) under three conditions: Acc1—the maximum accuracy achieved actually; Acc2—the minimum accuracy achieved actually; Acc3—the accuracy yielded by TL-LEDA-TSS algorithm.

TS	1	2	3	4	5	6	7	8	9	10	11	12	13	14	15	16	M
Acc1	100 (2)	100 (7)	100 (15)	97.5 (1)	82.5 (4)	100 (1)	90 (15)	90 (9)	100 (6)	97.5 (15)	88.75 (7)	100 (8)	75 (5)	86.25 (15)	91.25 (10)	62.5 (1)	91.33 (7.56)
Acc2	98.75 (1)	98.75 (1)	95 (3)	92.5 (10)	75 (1)	95 (15)	75 (2)	70 (2)	97.5 (8)	92.5 (14)	83.75 (2)	95 (1)	65 (12)	65 (2)	87.5 (1)	62.5 (1)	84.3 (4.75)
Acc3	100 (13)	100 (9)	98.75 (9)	97.5 (4)	81.25 (6)	98.75 (11)	91.25 (8)	87.5 (9)	100 (7)	97.5 (8)	90 (8)	100 (8)	72.5 (5)	82.5 (10)	86.25 (8)	62.5 (7)	90.39 (8.13)

The digits in parentheses denote the corresponding number of source subjects.

At all numbers of training trials, the accuracies of TL-LEDA-ASS are higher than those of SSL and the accuracies of TL-LEDA-TSS are higher than those of TL-LEDA-ASS, meaning that both data alignment and subject selection promoted the performance of transfer learning. The fewer the training trials, the greater is the performance improvement. Especially when the number of training trials is 5, the accuracies of TL-LEDA-TSS and TL-LEDA-ASS are 13.6% and 11.33% higher than that of SSL respectively. This makes sense for transfer learning because its goal is to decrease calibration time. Based on paired t-tests, statistically significant differences in accuracy (*p* values) among the four algorithms at 95% confidence level are shown in Table 2.

**Figure 9:**
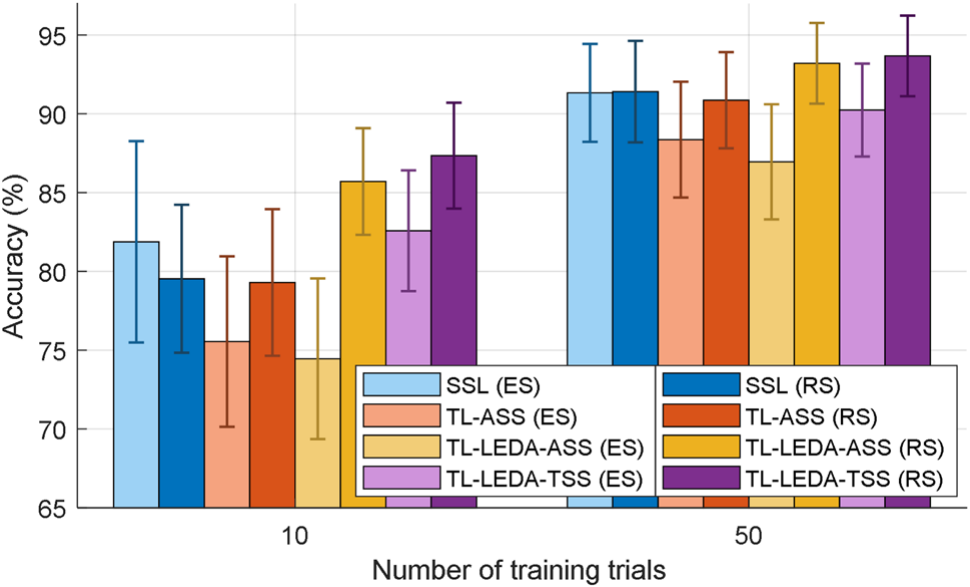
Averaged classification accuracies across subjects yielded by the two groups of algorithms derived from Euclidean and Riemannian space respectively in the cases of 10 and 50 training trials. ES and RS denote Euclidean space and Riemannian space respectively. The error bars represent the standard error.

(5) Riemannian space vs Euclidean space: Fig. 9 shows the comparison of averaged classification accuracies across subjects yielded by the two groups of algorithms developed in Euclidean and Riemannian space respectively in the cases of 10 and 50 training trials. It is clearly observed from the figure that for the two numbers of training trials, the subject-specific learning algorithm from Euclidean space performs better than or equal to that from Riemannian space, whereas the three transfer learning algorithms from Riemannian space are superior to those from Euclidean space. Using the subject-specific algorithm from Euclidean space (SSL (ES)) as a baseline, the two transfer learning algorithms from Riemannian space (TL-LEDA-ASS (RS) and TL-LEDA-TSS (RS)) still improved the classification performance, especially when the number of training trials is small. For all 10 numbers of training trials, statistically significant difference in accuracy (*p* values) between each pair of algorithms derived from Riemannian and Euclidean space based on paired t-tests at 95% confidence level is listed in Table 3.
(6) Computational complexity: As shown in Fig. 1, the classification process of this proposed algorithm TL-LEDA-TSS includes two phases of training and testing. In the training stage, the algorithm contains the five procedures of aligning data, creating super-trials, estimating SCMs, selecting source subjects and computing log-Euclidean mean per class; In the testing stage, the algorithm contains four procedures, the first three of which are the same as those in training stage, whereas the last one is to classify single-trial data based on the MDM classifier. The training stage is long because the shrinkage approach is used to estimate SCMs and numerous trials are required for training, whereas the testing stage is much shorter because data processing in online experiments is done trial by trial. The evaluation process was carried out on a computer with the configuration of Intel (R) Core (TM) i5-6500 CPU @ 3.20 GHz, 8.00 GB RAM and 64-bit OS. Assume that 20 training trials are available from a target subject and all source subjects are acted as transferred ones, the training stage takes about 9.8 min. Fortunately, the log-Euclidean mean per class used for testing stage can be calculated offline. Classifying a single-trial EEG data takes only 0.75 s, which is suitable for online processing.

**Table 2:** Statistically significant differences in accuracy (*p* values) among the four algorithms based on paired t-tests at the confidence level of 95%.

NT	M1-M2	M1-M3	M1-M4	M2-M3	M2-M4	M3-M4
5	0.090	**0.001**	**0.0002**	**0.011**	**0.005**	0.294
10	0.938	**0.017**	**0.003**	**0.011**	**0.010**	0.160
15	0.212	0.126	**0.002**	**0.016**	**0.005**	0.056
20	0.186	0.119	**0.006**	**0.026**	**0.013**	0.109
25	0.250	0.229	**0.019**	**0.032**	**0.009**	0.123
30	0.252	0.508	**0.046**	0.052	**0.010**	0.135
35	0.634	0.291	**0.032**	0.078	**0.014**	0.357
40	0.832	0.070	**0.013**	**0.022**	**0.007**	0.094
45	0.800	0.052	**0.012**	**0.030**	**0.009**	0.237
50	0.531	0.070	**0.021**	**0.015**	**0.009**	0.211

NT: Number of training trials; M1: SSL; M2: TL-ASS; M3: TL-LEDA-ASS; M4: TL-LEDA-TSS.

Significant values are in bold.

**Table 3:** Statistically significant difference in accuracy (*p* values) between each pair of algorithms developed in Riemannian and Euclidean space respectively based on paired t-tests at 95% confidence level, at 10 numbers of training trials.

Number of trials	5	10	15	20	25	30	35	40	45	50
SSL	**0.002**	0.469	**0.011**	**0.013**	**0.045**	0.054	0.116	0.052	0.155	0.876
TL-ASS	0.287	0.277	0.327	0.354	0.243	0.202	0.101	0.159	0.098	0.075
TL-LEDA-ASS	**0.004**	**0.003**	**0.002**	**0.001**	**0.001**	**0.002**	**0.004**	**0.003**	**0.002**	**0.003**
TL-LEDA-TSS	**0.033**	**0.037**	**0.016**	**0.016**	**0.007**	**0.035**	**0.045**	**0.010**	**0.011**	**0.003**

Significant values are in bold.

## Discussion

A typical c-VEP BCI system requires a training stage to obtain sufficient calibration data. However, this cumbersome training procedure will limit its practical application. How to reduce the training time without sacrificing the accuracy is one of the main research directions. The results in this study indicated that compared to the subject-specific (baseline) algorithm, the transfer learning algorithm incorporating LEDA and TSS significantly increased the classification accuracy at each given number of training trials. In other words, the proposed algorithm achieved the similar accuracy to the baseline algorithm using much fewer training trials, and thus reduced the training time substantially.

The experimental results show that TL-LEDA-ASS is better than TL-ASS, and the TL-LEDA-TSS is better than TL-LEDA-ASS. These findings prove that both data alignment and source subject selection can improve transfer learning. At the same time, both TL-LEDA-TSS and TL-LEDA-ASS are better than SSL especially in the case of fewer training trials, where the improvement in accuracy is more obvious. These conclusions support our goal to reduce training time while maintaining high accuracy.

In the previous studies on c-VEP BCI, we did some meaningful work. We first made a study on stimulus specificity of c-VEP BCIs^55^. Five experiments were devised to investigate the effect of size, color and proximity of the stimuli, length of modulation sequence and the lag between two adjacent stimuli on target recognition. An optimal value of each parameter was attained in terms of classification performance, and thus provides a basis for designing a high-performance c-VEP BCI. To make BCI suitable for complex applications like word input, we then presented a novel c-VEP BCI paradigm for increasing the number of stimulus targets based on grouping modulation with different codes^17^. Using the paradigm, a BCI with 48 targets divided into three groups was implemented with a high ITR of 181 bits/min. Finally, we further expended the paradigm to four target groups and implemented a c-VEP BCI of 64 stimulus targets with an ITR of 184.6 bit/min^56^. All these methods were developed in Euclidian space. In this study, we developed a Riemannian geometry-based classification frame for c-VEP BCIs.

As mentioned in section Introduction, the methodologies of Riemannian geometry have been successfully applied to BCIs based on MI, f-VEP and P300. For MI-based BCIs, Riemannian geometry is mainly used for matching the statistical distributions of two datasets so that transferring data from source subjects to a target subject becomes effective. Zanini et al.^47^ proposed a RA approach that aligns the covariance matrices of a subject with the reference matrix estimated by the EEG data of resting states, i.e., the transitional periods between two trials. Rodrigues et al.^24^ presented a Procrustes analysis-based method for aligning two data sets using geometrical transformations (translation, scaling and rotation) over the data points. Tang et al.^26^ proposed a Riemannian mean-based rotation alignment (RMRA) domain adaptation method by rotating the SPD matrix in Riemannian space. Another study conducted by Kalaganis et al.^25^ presented a method called discriminative covariance reduction (DCR) to reduce the dimensionality of covariance matrices by turning the problem of sensor selection as a maximization of a function over the manifold of SPD matrices. In terms of f-VEP-based BCIs, Kalunga et al.^27^ conducted the first study of online classification based on Riemannian geometry, in which they proposed a novel algorithm for asynchronous processing of brain signals, borrowing the principles of from semi-unsupervised approaches and following a dynamic stopping scheme to provide a prediction as soon as possible. In another study, Kalunga et al.^28^ presented a transfer learning method for SSVEP-based BCIs by making use of available data from previous users, relying on Riemannian geometry to estimate the similarity with a muti-user dataset and borrowing the notion of composite mean to partition the space. As for P300-based BCI, Li et al.^29^ proposed a transfer learning algorithm that combines XDAWN spatial filter and Riemannian geometry classifier. The XDAWN is used to enhance the P300 component in the raw signal as well as reduce its dimensions. The Riemannian mean is acted as the reference matrix to perform the affine transformation of SPD matrices that makes the data from different subjects comparable.

When Riemannian geometry methodologies are applied to c-VEP based BCIs, the dimensionality of covariance matrices is $N_{c} (Z + 1) \times N_{c} (Z + 1)$ as shown in Eq. (3), which is high when both the number of channels $N_{c}$ and the number of stimulus targets $Z$ are large, resulting in high computational complexity of class means. To address the problem, a method for channel selection is usually necessary to select a small number of discriminative channels for given brain tasks. Recently, Jin et al. proposed two methods^57,58^ for improving the classification performance of MI-based BCIs. They are based on the assumption that the channels related to MI should contain common information when participants are executing the same tasks. One method is called the correlation-based channel selection (CCS) and the other is named bispectrum-based channel selection (BCS). The former aims to select task-related channels and meanwhile exclude those channels containing redundant information and noise. A novel regularized CSP is used to extract effective features and an SVM classifier with the radial basis function is trained to accurately identify MI tasks; The latter aims at extracting non-linear and non-Gaussian information from EEG signals with bispectrum analysis. The method uses the sum of logarithmic amplitudes (SLA) and the first-order spectral moment (FOSM) to select EEG channels without redundant information. In the previous study on electrocorticogram (ECoG)-based MI BCIs, we proposed a wrapper method for selecting a suitable number of task-related channels to increase the discriminability of feature signals^59^. CSP algorithm was used for feature extraction, and channel selection was performed by genetic algorithm (GA) for optimizing the feature extraction. Fisher discriminant analysis (FDA) was used as the classifier, and the channel subset chosen at each generation was evaluated by classification accuracy. As for the current study, a channel selection algorithm can decrease the number of channels and resulting computational complexity in the estimation of SCMs, but this is beyond the scope of this study.

This study evaluated the proposed algorithm offline and applied it to a c-VEP BCI containing 16 stimulus targets. The effectiveness of the proposed algorithm is verified in terms of reducing training time. One limitation of the proposed algorithm is that the dimensionality of super-trials increases rapidly with the number of stimulus targets, leading to long time for estimating SCMs with the shrinkage approach and the consequent difficulty in estimating class means. Another limitation is that all subjects participated in the study are healthy ones without the involvement of the people with severe neural impairments. Future work will focus on generalizing the algorithm to c-VEP BCIs with more stimulus targets, experimenting on subjects with nerve injury and its online implementation.

## Conclusion

This paper proposes a transfer learning algorithm of c-VEP BCIs based on Riemannian geometry, which combines data alignment and subject selection to improve the performance of transfer learning. In the proposed algorithm, log-Euclidean data alignment (LEDA) is employed to reduce the difference in data distribution between subjects, whereas training accuracy-based subject selection (TSS) is utilized to select out the source subjects with higher similarity to the target subject. A comprehensive off-line analysis was conducted on the experimental data derived from 16 subjects. The results indicated that compared to the subject-specific algorithm, the proposed transfer leaning algorithm effectively reduces the training time of the c-VEP BCI at the same performance level, and thereby facilitates its application in real world.

## Data Availability

The data can be made available upon reasonable request by contacting the corresponding author.
